# A simple, highly efficient *Agrobacterium tumefaciens*‐mediated moss transformation system with broad applications

**DOI:** 10.1007/s42994-024-00174-4

**Published:** 2024-07-19

**Authors:** Ping Zhou, Xiujin Liu, Yuqing Liang, Yan Zhang, Xiaoshuang Li, Daoyuan Zhang

**Affiliations:** 1 State Key Laboratory of Desert and Oasis Ecology, Key Laboratory of Ecological Safety and Sustainable Development in Arid Lands, Xinjiang Institute of Ecology and Geography, Chinese Academy of Sciences, Urumqi, 830011 China; 2 Department of Food Science and Engineering, Moutai Institute, Renhuai, 564502 China; 3 Present Address: Xinjiang Key Lab of Conservation and Utilization of Plant Gene Resources, Xinjiang Institute of Ecology and Geography, Chinese Academy of Sciences, Urumqi, 830011 China; 4 University of Chinese Academy of Sciences, Beijing, 100049 China; 5 Shanghai Center for Plant Stress Biology, CAS Center for Excellence in Molecular Plant Sciences, Chinese Academy of Sciences, Shanghai, 200032 China

**Keywords:** *Agrobacterium tumefaciens*, Highly efficient, Moss, Transformation

## Abstract

Mosses, particularly desiccation-tolerant (DT) species, are important model organisms for studying genes involved in plant development and stress resistance. The lack of a simple and efficient stable moss transformation system has hindered progress in deciphering the genetic mechanisms underlying traits of interest in these organisms. Here, we present an *Agrobacterium tumefaciens*-mediated transformation system for DT mosses that uses *Agrobacterium* strain EHA105 harboring the binary vector pCAMBIA1301-GUS. This system achieved transformation efficiencies of 74% and 81% in *Physcomitrium patens* and *Bryum argenteum* protonemata, respectively, without the need for culture and callus formation prior to regeneration. We detected GUS enzyme activity in the regenerated transgenic moss via histochemical staining. Southern blot, PCR, and RT-qPCR analyses confirmed the presence of the *GUS* gene. In addition, we successfully used this system to transform wild DT *Syntrichia caninervis*. Furthermore, *P. patens* and *B. argenteum* transformed using this system with the stress resistance gene *EsDREB* from the desert plant *Eremosparton songoricum* (Litv.) exhibited improved salt tolerance. We thus present an efficient tool for the genetic analysis of DT moss species, paving the way for the development of stress-resistant crop cultivars.

**Supplementary Information:**

The online version contains supplementary material available at 10.1007/s42994-024-00174-4.

## Introduction

As a close relative of early vascular plants, the model moss *Physcomitrium patens* is a useful tool for bioinformatics and molecular biology studies of plant development and stress resistance. The *P. patens* genome resembles that of *Arabidopsis thaliana* in its size and high frequency of homologous recombination, but resembles that of yeast in its inefficiency of gene targeting and haploid-dominant life cycle. These features make *P. patens* an interesting and useful tool for molecular genetic studies in plants (Lang et al. 2008; Schaefer 2002; Schuette et al. 2015, 2009). The most common methods to transform this moss are through particle bombardment and polyethylene glycol (PEG)-mediated DNA uptake, specifically PEG-mediated transformation of individual cells that are then regenerated into whole plants (Rensing et al. 2020). Although many species have been transformed in this manner, these methods remain challenging, particularly for *P. patens*, because they rely on the moss protonemata being at a specific stage of differentiation. Moreover, it is challenging and time consuming to extract protoplasts from protonemata and to manage their subsequent differentiation (Newell 2000).

Upon rehydration, desiccation-tolerant (DT) bryophytes can fully recover after losing virtually all their free intracellular water (Proctor et al. 2007). The DT moss *Bryum argenteum*, an important biotic component of ecosystems in northwestern China (Zhang et al. 2007), is emerging as a useful organism for studying the molecular, structural, and ecological aspects of vegetative desiccation tolerance in plants (Gao et al. 2015; Li et al. 2014a; Stark et al. 2010). The DT moss *Syntrichia caninervis* Mitt. (Pottiaceae), a biotic component of North American and Asian dryland ecosystems (Silva et al. 2021; Stark and Brinda 2015), has also emerged as a model plant for studying desiccation tolerance, dehydration, and rehydration (Buitink et al. 2000; Gao et al. 2014). Genetic transformation systems have not been developed for *B. argenteum* or *S. caninervis*, limiting the analysis of gene function in DT mosses and, more generally, evolutionary developmental biology studies of land plants.

*Agrobacterium tumefaciens*-mediated transformation has long been used for the genetic manipulation of a broad variety of plants, because it does not require expensive equipment and leads to the stable expression of foreign genes (Takata and Eriksson 2012). *Agrobacterium*-mediated stable transformation can yield high expression of target genes in monocotyledons, such as rice (*Oryza sativa*) (Raineri et al. 1990) and maize (*Zea mays*) (Ishida et al. 1996), dicotyledons, such as *A. thaliana* (Zhang et al. 2006), and the liverwort *Marchantia polymorpha* (Tsuboyama and Kodama 2014), but this method has not yet been reported in moss. This approach has several advantages over other transformation methods, such as the ability to integrate a small number of transgene copies into the plant genome and to transfer relatively large DNA segments and integrate intact transgenes into the genome (Frangedakis et al. 2021).

In this study, we developed the first successful *Agrobacterium*-mediated transformation system for DT mosses. Our method is based on the *Agrobacterium*-mediated transformation of protonemata or gametophytes. We first optimized our protocol for mosses using protenemata as starting material because of their rapid growth and then extended the method to gametophytes from the wild moss *S. caninervis*. The transformants regenerated directly from transformed gametophytes without the need for culture and callus formation prior to regeneration. Finally, we introduced an abiotic stress-resistance gene from *Eremosparton songoricum* (Litv.) (*EsDREB*), a flowering pioneer plant adapted to desert environments that is used in the Gurbantunggut Desert of Xinjiang to protect the local ecosystem from desertification, into the two moss species to verify that the system can be used for the functional study of DT genes (Li et al. 2014b).

## Results

In our effort to develop a simple and reliable stable transformation system for mosses, we tested the potential of *Agrobacterium*-mediated gene delivery to generate stable transgenic moss lines. Several factors determine the efficiency of *Agrobacterium*-mediated gene delivery: the antibiotic concentration used to select transgenic lines, the preculture time before infection, the sucrose concentration in the growth medium, the *Agrobacterium* strain, and the infection period. We examined each of these factors in this study.

To achieve stable moss transformants, we used the pCAMBIA1301 vector containing the *GUS* reporter gene. We also used the pBI121-GFP overexpression vector for transformation of moss with *EsDREB*. This gene was chosen because *EsDREB2B* transcript accumulation is induced in *E. songoricum* by a variety of abiotic stresses, including drought, salinity, cold, heat, heavy metals, mechanical wounding, and oxidative stress. The transgenic gametophytes or protonemata obtained using this protocol can be cultivated in various media tailored to specific requirements, accommodating diverse starting materials and expediting the acquisition of transgenic material.

### Selection of the appropriate antibiotic concentration

**Fig. 1:**
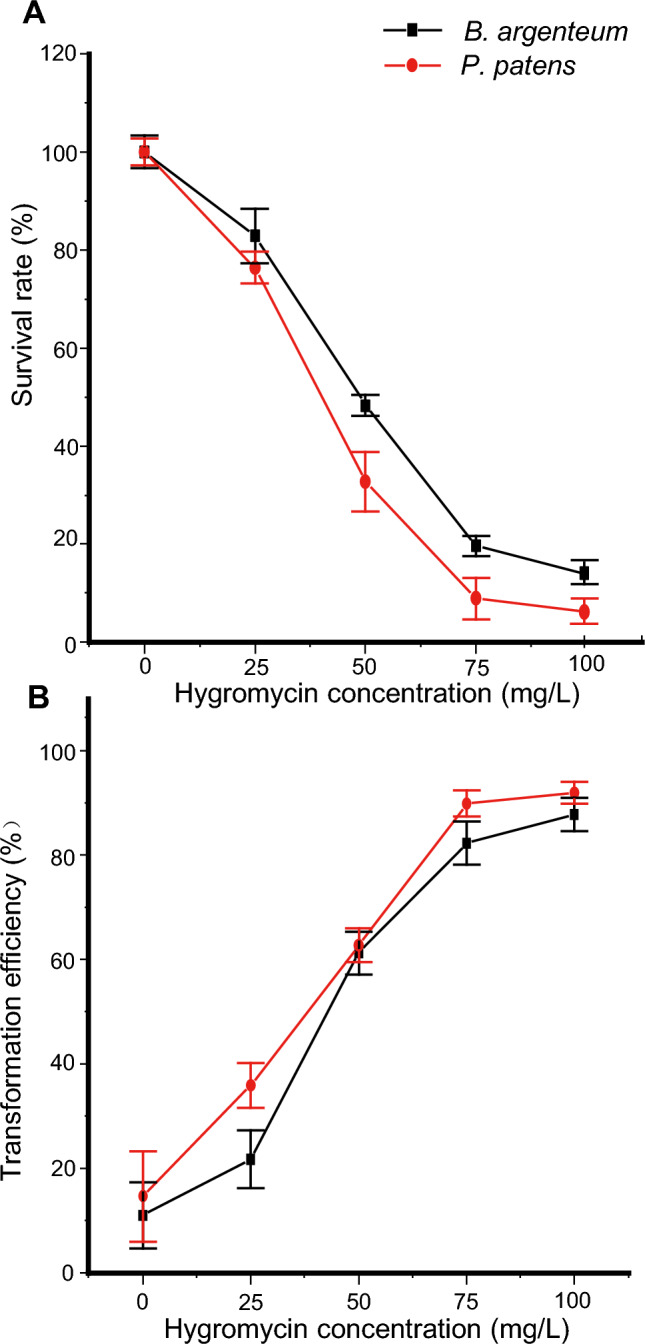
Recovery of stable transgenic *P. patens* and *B. argenteum* protonemata using hygromycin as the selectable marker. **A** Survival rate of moss protonemata incubated with the indicated hygromycin concentrations for 15 days. **B** Transformation efficiency of moss protonemata incubated with the indicated hygromycin concentrations for 15 days. Date are mean ± SEM (n = three independent sets of experiments)

A selectable marker gene is required for the efficient recovery of stable transgenic lines following *Agrobacterium* transformation. We chose the hygromycin resistance gene as the selectable marker gene. To identify the appropriate hygromycin concentration for *P. patens* and *B. argenteum*, we treated untransformed *P. patens* and *B. argenteum* protonemata with concentrations ranging from 0 to 100 mg/L. A 3-week incubation with 75 mg/L hygromycin was sufficient to inhibit the growth of untransformed protonemata of both *P. patens* and *B. argenteum*, with survival rates of 9% and 20%, respectively (Fig. 1A). The sensitivity of the protonemata to hygromycin did not differ appreciably between the two mosses.

We also tested the antibiotic concentrations on transformed plants, which we produced using *Agrobacterium* strain EHA105, media with no sucrose, preculture of 6 days, *Agrobacterium* antibiotic of OD_600_ = 0.6, 30 min infection time, and a transformation duration of 30 min. The transformation efficiency did not significantly increase at a hygromycin concentration of 100 mg/L compared to 75 mg/L (Fig. 1B). Thus, we selected 75 mg/L as the appropriate hygromycin concentration.

### Selection of transgenic plantlets

**Fig. 2:**
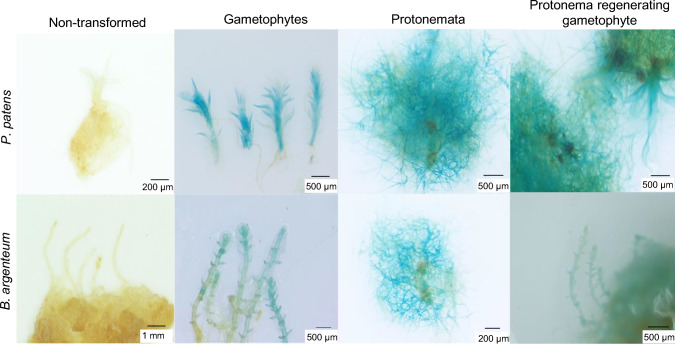
Histochemical assay of *GUS* expression in moss transformed with *Agrobacterium* harboring pCAMBIA1301-GUS

For each transformation, we added hygromycin screening solution (75 mg/L) to *P. patens* and *B. argenteum* protonemata in a culture dish, cultured them for 1 month, and then selected healthy protonemata for further investigation (Fig. S1). To confirm the successful integration of the transgene into the surviving protonemata, we performed GUS staining to examine GUS activity throughout the moss life cycle (Fig. 2). GUS activity was observed in the selected protonemata, gametophytes, and regenerated gametophytes.

For plants that tested positive for GUS activity, we confirmed the integration of the transgene through PCR amplification of the *GUS* gene followed by DNA purification and gel electrophoresis. The expected band size of 823 bp from amplification of a fragment of the *GUS* gene was amplified from the DNA derived from the regenerated plant lines (Fig. S2). These results demonstrated that the regenerated lines indeed represented transgenic plants.

**Fig. 3:**
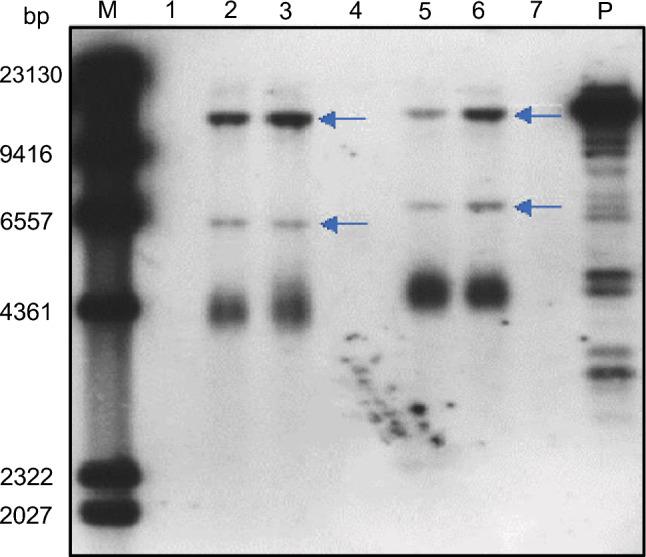
Southern blot analysis of transgenic *P. patens* and *B. argenteum* with *GUS* gene. M: DNA size markers; P: pCAMBIA1301-GUS, positive control; 2, 3: transformed *P. patens*; 5, 6: transformed *B. argenteum*; 1, 4: digested DNA derived from non-transformed *P. patens* and *B. argenteum*, respectively; 7: blank control. The arrows indicate inserted copy of the *GUS* transgene

To confirm the stable integration of the transgene in PCR-positive plants, we performed Southern blot analysis. To this end, we digested genomic DNA with *Hin*dIII, which cuts once within the T-DNA, excising the *GUS* gene along with part of the genomic DNA at the integration site. Plants with a single integrated T-DNA should produce a single band. We detected the resultant fragment by hybridization to a 1,029-bp radiolabeled probe specific for the *GUS* gene from pCAMBIA-1301. Samples from both mosses contained the *GUS* transgene, thus confirming the stable integration of the transgene (Fig. 3). For the *GUS*-positive samples, digestion with *Hin*dIII resulted in two clear bands, indicating the presence of two copies of the T-DNA in the sample.

### Optimization of factors influencing* Agrobacterium*-mediated transformation

**Fig. 4:**
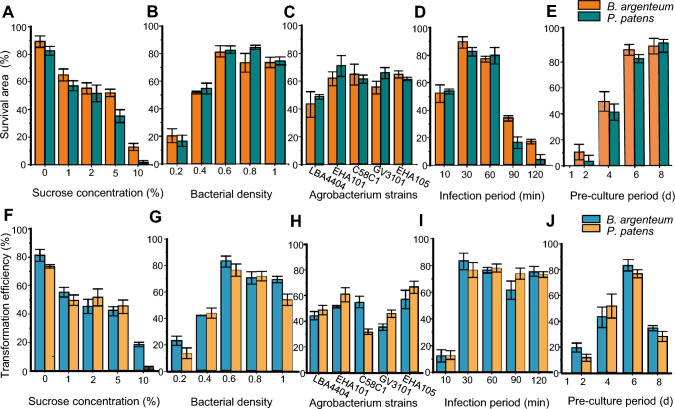
Effects of different factors on protonemata survival rate and transformation efficiency. **A** and **F** Survival rate and transformation efficiency of transformed *P. patens* and *B. argenteum* under different sucrose concentrations. **B** and **G** Survival rate and transformation efficiency using different *Agrobacterium* cell densities (OD_600_ values of 0.2, 0.4, 0.6, 0.8, and 1.0). **C** and **H** Survival rate and transformation efficiency using the indicated *Agrobacterium* strains. **D** and **I** Survival rate and transformation efficiency after the indicated infection periods. **E** and **J** Survival rate and transformation efficiency following the indicated pre-culture period. Each treatment comprised samples on five petri dishes and was performed in triplicate. Data are mean ± SD

We evaluated the effects of sucrose concentration, *Agrobacterium* cell densities, *Agrobacterium* strain, infection period, and duration of preculture, on the transformation efficiencies and survival rates of *P. paten*s and *B. argenteum* protonemata (Fig. 4). Following transformation, the mosses were grown on selection medium, for 30 days, to identify transformants, allowing us to calculate their survival rates and transformation efficiencies.

Sucrose concentrations of 0%, 1%, 2%, 5%, and 10% in the transformation buffer resulted in median *B. argenteum* transformation efficiencies of 81%, 55%, 45%, 42%, and 18%, and median *P. patens* transformation efficiencies of 74%, 50%, 52%, 45%, and 17%, respectively (Fig. 4A). Thus, including sucrose in the transformation buffer decreased the transformation efficiency in a dose-dependent manner. Indeed, sucrose in the medium led to the browning of the moss plants and the entrance of *Agrobacterium* into the transition growth phase, which would be expected to reduce the transformation efficiency.

The amounts of *Agrobacterium* relative to moss protonemata, the *Agrobacterium* strain, and the infection period also influenced the transformation efficiency. For *B. argenteum*, we observed a significant increase in transformation efficiency with increasing *Agrobacterium* cell densities from OD_600_ 0.1 to 0.6, followed by a subsequent significant decrease in efficiency and the overgrowth of *Agrobacterium* at OD_600_ > 0.6 (Fig. 4B). *P. patens* showed a similar trend, but with the peak transformation efficiency seen at an *Agrobacterium* OD_600_ of 0.8. Among the five *Agrobacterium* strains tested, EHA105 exhibited the highest transformation efficiency for both *B. argenteum* and *P. patens* (Fig. 4C). In terms of the infection period, either an increase or a decrease from our original protocol of 30 min significantly decreased the percentage of surviving moss protonemata in both moss species (Fig. 4D).

We also investigated whether preculturing the moss affected the transformation efficiency. Preculturing the protonemata on PpNH4 medium for 1, 2, 4, 6, or 8 days, in a petri dish sealed with cellophane led to median *B. argenteum* transformation efficiencies of 0%, 19%, 44%, 81%, and 77%, and median *P. patens* transformation efficiencies of 0%, 12%, 51%, 74%, and 58%, respectively (Fig. 4E). These results indicated that a preculture period of 6 days was optimal.

### Testing transgene inheritance

We next assessed whether the transgene could be stably inherited into moss throughout its life cycle. Following regeneration of the transgenic lines, the generation of gametophytes and the expression of the *GUS* gene in the regenerated gametophytes indicated that the transgene had been successfully passed on to the next generation. Moreover, we tested transgene inheritance for at least 10 generations (Fig. S3) and determined that it was still stably inherited.

### Application of the transformation system to other mosses

**Fig. 5:**
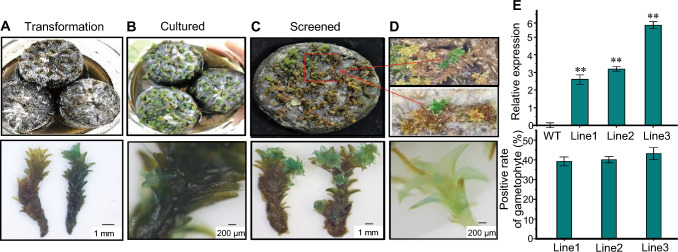
Histochemical assay of *GUS* expression in *S. caninervis* transformed using *Agrobacterium*. **A,** **B** Phenotype and histochemical staining was performed after 0 and 7 days of transformation, respectively. **C** Phenotype and GUS staining was performed after a 15 day selection period.** D** Phenotype and GUS staining in regenerated gametophytes. **E** Statistical analysis of relative *GUS* expression levels and regenerated gametophytes positivity rate. Data are mean ± SD of three biological replicates; asterisks indicate significant differences from WT (**P < 0.01, Student’s *t* test)

To determine whether our method could be used for transformation of other moss species, we tested it on the typical desert moss, *S. caninervis.* Seven days after transformation, we detected GUS activity in the *S. caninervis* gametophytes, and after 7 days of co-culture, we also detected GUS activity in the regenerated moss plants. After 15 days of continuous selection, the T_1_ plants were green, and subsequent GUS assays yielded blue-stained regenerated moss plants (Fig. 5A–D). The final percentage of GUS-stained plants among the regenerated plants was 45%. The relative expression level of *GUS* was detected in the transgenic lines (Fig. 5E). These results demonstrated that this method can be successfully applied to *S. caninervis* and that the transgene can be stably inherited.

### Engineering stress tolerance in mosses

**Fig. 6:**
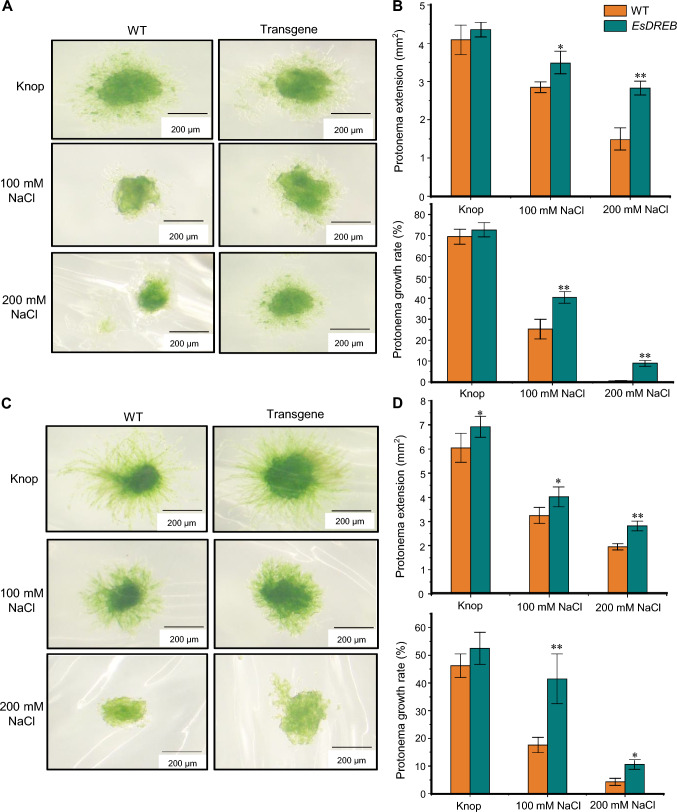
Functional verification of *EsDREB* in salt stress tolerance through heterologous expression in *P. patens* and *B. argenteum*. **A** Growth phenotype of *P. patens*. **B** Protonemata extension and growth rate of *P. patens*. **C** Growth phenotype of *B. argenteum*. **D** Protonemata extension and growth rate of *B. argenteum*. Protonemata growth rate = (growth area of protonemata before treatment)/(growth area of protonemata after treatment) × 100%. Each treatment comprised samples on five petri dishes and was performed in triplicate. Data are mean ± SD (each treatment comprised five petri dishes and was performed in triplicate)

To further assess our stable transformation method, we used it to introduce the stress resistance gene *EsDREB* into *P. patens* and *B. argenteum*, which we then treated with different salt concentrations (100 and 200 mM NaCl). In both species, the growth rates and extension of protonemata produced by these transgenic mosses, after 30 days of growth on PpNH4 moss growth medium (without added NaCl), were greater than those for wild-type *P. patens* and *B. argenteum* (Fig. 6). When grown in the presence of 100 mM NaCl, the protonemata of both the transgenic and wild-type mosses had a smaller growth area than those grown in the absence of added NaCl, but the areas of the transgenic protonemata were significantly larger than those of the wild-type mosses. Treatment with 200 mM NaCl had similar, but stronger, effects on growth, with the protonemal growth areas of the transgenic mosses again being significantly greater than those of the wild-type plants of each species. These results indicated that the *EsDREB* transgene improves the growth of moss protonemata under salt stress and confers salt stress tolerance. Therefore, our method can be successfully used to verify the function of genes from other plants.

## Discussion

*Agrobacterium*-mediated transformation has been used in various plant species (Frangedakis et al. 2021; Nishimura et al. 2006; Olhoft et al. 2004). Traditional *Agrobacterium*-mediated genetic transformation requires the establishment of tissue culture systems, which are time-consuming and result in low transformation efficiencies. Although several transformation methods not requiring tissue culture have recently been described, they are not compatible with all types of plants (Cao et al. 2023a, b).

An *Agrobacterium*-mediated stable transformation method has not previously been established for moss. Historically, gene targeting was accomplished in *P. patens* through PEG-mediated transformation of protoplasts, a method widely used in plants. For more efficient gene manipulation in *P. patens*, RNA interference, CRISPR/LbCas12a, and particle bombardment methods have been developed and applied (Pu et al. 2019; Bezanilla et al. 2003; Cho et al. 1999). However, genetic transformation systems for DT mosses, such as *S. caninervis*, *B. argenteum*, and *Syntrichia ruralis*, have not been reported. This lack of molecular genetic methods for mosses limits studies of DT mosses and the exploration and utilization of genes conferring desiccation tolerance.

### Comparing PEG- and *Agrobacterium*-mediated transformation methods

We compared the outcomes of classical transformation (PEG-mediated transformation of protoplasts) with those of *Agrobacterium*-mediated transformation in *P. patens*. In PEG-mediated transformation, protonemata cultured for 6 days are processed into protoplasts, which are subsequently transformed and screened, and transgenic protonemata are obtained within 10–50 days with a transformation efficiency of 0.5–1 transformants/µg DNA. This technique yields ~ 10^6^ protoplasts per 90-mm-diameter plate of tissue, with relative transformation frequencies of 1 to 22 integrative transformants per 10^5^ regenerated plants, depending on the vector (Cove et al. 2009; Schaefer and Zrÿd 1997). Thus, a 90-mm culture dish can produce up to 22 transformants following protoplast transformation.

In our *Agrobacterium*-mediated transformation method, protonemata cultured for 6 days are directly transformed and subsequently screened, resulting in transgenic lines within 37 days. We obtained a positive transformation rate of 73%, which should yield an even greater number of transgenic protonemata. The duration required to obtain transgenic protonemata differs only slightly between the two methods. However, *Agrobacterium-*mediated transformation is significantly more efficient than PEG-mediated transformation, as the former directly targets protonemata, enabling the rapid generation of a substantial number of transgenic strains. In addition, protonemata can be transferred to Knop medium during transformation, allowing the direct acquisition of transgenic gametophytes (Fig. S4).

### Optimization of factors influencing *Agrobacterium*-mediated transformation

In the current study, a straightforward, effective *Agrobacterium*-mediated transformation protocol was devised for mosses using protonemata or gametophytes. In our approach, *Agrobacterium* directly infects protonemata or gametophytes, eliminating the need for protoplast preparation or regeneration to yield intact transgenic plants. To improve the efficiency of moss transformation, we optimized several factors, including the sucrose concentration, *Agrobacterium* cell concentration, bacterial strain, infection duration, and preculture duration.

The *Agrobacterium* cell densities used is a critical factor influencing transformation efficiency (Amoah et al. 2001). Elevated bacterial cell density may result in the overgrowth of *Agrobacterium* on the medium, thereby impeding the survival of mosses and diminishing the transformation efficiency. Conversely, lower concentrations of *Agrobacterium* can lead to reduced transformation efficiency. The optimal transformation density of *Agrobacterium* varies based on the species and strain of *Agrobacterium* used. For instance, for *Marchantia polymorpha*, an *Agrobacterium* density corresponding to OD_600_ = 0.5 might already be saturating for the transformation of sporelings (Tsuboyama and Kodama 2014). In our method, the *Agrobacterium* density yielding the highest transformation efficiency was an OD_600_ of 0.6.

The infection duration affects transformation efficiency (Manders et al. 1994). Here, we obtained the greatest transformation efficiency with an incubation period of 30 min. The *Agrobacterium* strain used also affects plant transformation efficiency (Azizi-Dargahlou and Pouresmaeil 2023; Frangedakis et al. 2021), which we confirmed in our system, finding that strain EHA105 exhibited the highest transformation efficiency in both moss species.

The state of the plant material also affects the transformation efficiency, as the plant susceptibility to *Agrobacterium* infection varies at different stages of culture. In *M. polymorpha*, preculture for 7 days led to the highest transformation efficiency (Ishizaki et al. 2008). In the present study, the most effective period of protonemal preculture was 6 days. Furthermore, the optimal infection duration varies by plant species (Jha et al. 2011; Kumar et al. 2019; Yang et al. 2016). When the infection duration is too short, the probability of exogenous DNA insertion into the moss genome is low, and when the infection duration is too long, destruction of protonemata can easily occur. We established that, for the mosses we studied, the transformation efficiency was greatest after 30 min of infection, with shorter or longer periods being less effective.

### Application of the transformation system to other mosses

Both *B. argenteum* and *S. caninervis* are DT mosses that lack established transformation systems. The absence of established transformation systems hinders studies of gene function in these species. We applied our transformation system to field materials of *S. caninervis* and directly obtained regenerated T_1_ generation gametophytes via re-culturing in peat pellets (Liu et al. 2021). Moreover, heterologous expression of a gene that functions in stress resistance in mosses resulted in enhanced salt stress resistance. Therefore, our method can be used to verify gene function in mosses.

In summary, we developed a rapid and simple transformation method for DT mosses using *Agrobacterium* strain EHA105. Optimized transformation conditions were as follows: the bacterial density was OD_600_ = 0.6; samples were precultured for 6 days; 200 μM acetosyringone was used as an inducer; and transformants were selected using 75 mg/L hygromycin. Under these optimized conditions, the transformation efficiency was almost 80%. Stable transgenic plants that expressed *GUS* were regenerated from protonemata or gametophytes. *Agrobacterium*-mediated transformation of protonemata or gametophytes enables the rapid generation of a relatively large number of transgenic plants and is amenable to scaling up. Moreover, this transformation system is well suited for transforming DT field moss materials, establishing a crucial foundation for identifying candidate genes associated with stress tolerance.

## Materials and methods

### Plant materials and treatments

*B. argenteum* and *P. patens* gametophytes were grown on Knop medium for 20 days and transformed as described below. *P. patens* and *B. argenteum* protonemata were grown for 1, 2, 4, 6, or 8 days on solid PpNH4 medium prior to transformation (Lopez-Obando et al. 2021).

### Plasmid construction and *Agrobacterium* culture

The plant binary vector pCAMBI1301 was introduced into *Agrobacterium* strains EHA105, LBA4404, GV3101, EHA101, and C58C1 via the freeze/thaw method.

A modified version of the AGROBEST protocol was performed, which involves antibiotic treatment of *Agrobacterium* cultures for 3 days followed by a 2 day co-culture period with moss (Wu et al. 2014). A single *A. tumefaciens* colony from a fresh plate was inoculated into 5 mL liquid LB medium supplemented with 50 mg/L kanamycin and 25 mg/L rifampicin (Solarbio, China) and incubated with shaking (220 rpm) at 28 °C for 20–24 h. To increase the number of *Agrobacterium* cells for the second activation, 1 mL of the bacterial suspension was inoculated into 50 mL of LB liquid medium supplemented with the appropriate antibiotics and incubated with shaking (220 rpm) at 28 °C for 12–16 h. The *Agrobacterium* cells were collected by centrifugation, resuspended in 50 mL AB:MES salts (17.2 mM K_2_HPO_4_, 8.3 mM NaH_2_PO_4_, 18.7 mM NH_4_Cl, 2 mM KCl, 1.25 mM MgSO_4_, 100 μM CaCl_2_, 10 μM FeSO_4_, 50 mM MES, 2% glucose [w/v], and 200 μM acetosyringone, pH 5.5), and grown overnight. Before being incubated with moss, the culture was again centrifuged and resuspended to an OD_600_ of 0.2, 0.4, 0.6, 0.8, or 1.0 in a 50:50 (v/v) mixture of AB:MES salts and half-strength Knop liquid moss growth medium (pH 5.5).

### *Agrobacterium*-mediated moss transformation

To simplify the *B. argenteum* and *P. patens* protonemal infection process, 5 mL of *Agrobacterium* culture was directly added to each culture dish. After 30 min of treatment, the bacterial solution was removed with a sterile pipette, and the culture plate was sealed with Parafilm. For gametophyte transformation, the gametophytes were placed in a 50-mL centrifuge tube, 20 mL of infection solution was added to the tube, and the mixture was returned to the culture plate after 30 min. The culture plates were sealed with cellophane. The plates were cultured in the dark for 3 days, and the cultures were transferred to standard culture conditions for *P. patens* for further cultivation. One month after screening and culture, validation was carried out.

For *S. caninervis* transformation, moss cultivated on peat pellets was directly inoculated by immersion for 30 min and then placed back onto the peat pellets. After 3 days of cultivation in the dark, the same light conditions as used for *P. patens* were resumed. Transformants were selected after 15 days of cultivation.

### Culture screening

After 7 days of co-cultivation, 2 mL of sterile water supplemented with 75 mg/L hygromycin was added to the moss culture dish. During the co-cultivation screening period, if *Agrobacterium* overgrowth occurred, the plants were washed twice with sterile water, after which screening solution was added.

For *S. caninervis* selection, *S. caninervis* cultivated on peat pellets was sprayed with distilled water containing 75 mg/L of hygromycin daily for 15 days.

### Plant RNA extraction and quantitative RT-qPCR

Total RNA was extracted from the samples using an EZNA Plant RNA Kit (OMEGA, USA). Total RNA (1 μg) was used to synthesize first-strand cDNA with One-Step gDNA Removal and cDNA Synthesis SuperMix (TransGen, China). Quantitative PCR was performed with 2 × SYBR Premix Ex Taq™ (TaKaRa, Japan). The *P. patens Actin* gene (AW698983) (Okada et al. 2009), *B. argenteum Actin* gene (TR77358|c0_g1_i4) (Gao et al. 2017), and *S. caninervis α-tubulin2* gene (Li et al. 2015) were used as internal controls.

### DNA extraction and PCR analysis

Genomic DNA was isolated from the samples using an EZNA® Plant DNA Kit (OMEGA, USA). *NPTII* (GenBank: AF234297.1) was used to verify T-DNA insertion events. The PCR primer set was designed to specifically amplify the DNA fragment of the *GUS* gene based on the nucleotide sequence of pCAMBIA1301 (accession number: AF234316). Phanta Max Super-Fidelity DNA Polymerase (Vazyme, China) was used according to the manufacturer’s instructions. The PCR procedure was as follows: initial denaturation at 95 °C for 30 s; 30 cycles at 95 °C for 10 s, 52 °C for 15 s, and 72 °C for 20 s; and a final extension step at 72 °C for 5 min.

### GUS staining assays

The transgenic protonemata or gametophytes were submerged in GUS staining solution containing 50 mM sodium phosphate (pH 7.0), 0.5 mg/L 5-bromo-4-chloro-3-indolyl-β-glucuronide, 0.1% Triton X-100, 0.5 mM K_3_[Fe(CN)_6_], and 0.5 mM K_4_[Fe(CN)_6_] overnight in darkness at 37 °C. The samples were washed several times in 75% ethanol to remove the chlorophyll (Jefferson et al. 1987). Tissues that turned blue harbored the *GUS* gene.

### Southern blot analysis

A 1-µg aliquot of genomic DNA was digested with *Hin*dIII at 37 °C. The digested DNA was subjected to electrophoresis on a 1% agarose gel and transferred to a Hybond-N^+^ nylon membrane (GE Healthcare) using a vacuum transfer device (BIO CRAFT). The DNA was hybridized with a radiolabeled probe comprising a random primer of a 1,052-bp fragment obtained by PCR amplification of pCAMBIA1301 with GUS primers (F: CGAAGTCACAGCCAAAAGCC; R: GCGAAATATTCCCGTGCACC).

### Statistical analysis

Statistical analysis of the survival rates and GUS staining frequencies of the mosses was performed by Student’s *t* test (*P* < 0.01). The survival rate of protonemata (%) = area of surviving protonemata/total area evaluated. The protonemal transformation efficiency (%) = (transgene-positive protonemal samples/surviving protonemata after screening × 100%). Each experiment was independently repeated three times.

## Supplementary Information

Below is the link to the electronic supplementary material.

Supplementary file1 (PDF 377 KB)

## Data Availability

All data generated during this study are included in the article and its supplementary information files.

## References

[CR1] Amoah BK, Wu H, Sparks C, Jones HD (2001) Factors influencing *Agrobacterium*-mediated transient expression of uidA in wheat inflorescence tissue. J Exp Bot 52:1135–1142. 10.1093/jexbot/52.358.1135 11432931 10.1093/jexbot/52.358.1135

[CR2] Azizi-Dargahlou S, Pouresmaeil M (2023) *Agrobacterium* tumefaciens-mediated plant transformation: a review. Mol Biotechnol. 10.1007/s12033-023-00788-x 37340198 10.1007/s12033-023-00788-x

[CR43] Bezanilla M, Pan A, Quatrano RS (2003) RNA interference in the moss *Physcomitrella patens*. Plant Physiol 133:470–474. 10.1104/pp.103.024901 14555775 10.1104/pp.103.024901 PMC523873

[CR3] Buitink J, Leprince O, Hoekstra FA (2000) Dehydration-induced redistribution of amphiphilic molecules between cytoplasm and lipids is associated with desiccation tolerance in seeds. Plant Physiol 124:1413–1426. 10.1104/pp.124.3.1413 11080316 10.1104/pp.124.3.1413 PMC59238

[CR4] Cao X et al (2023a) Cut-dip-budding delivery system enables genetic modifications in plants without tissue culture. Innovation (camb) 4:100345. 10.1016/j.xinn.2022.100345 36387605 10.1016/j.xinn.2022.100345 PMC9661722

[CR5] Cao X, Xie H, Song M, Zhao L, Liu H, Li G, Zhu JK (2023b) Simple method for transformation and gene editing in medicinal plants. J Integr Plant Biol 66(1):17–19. 10.1111/jipb.13593 10.1111/jipb.13593 38078380

[CR45] Cho SH, Chung YS, Cho SK, Rim YW, Shin JS (1999) Particle bombardment mediated transformation and GFP expression in the moss *Physcomitrella patens*. Mol Cells 9:14–19. 10.1016/S1016-8478(23)13501-1 10102565

[CR6] Cove DJ, Perroud PF, Charron AJ, McDaniel SF, Khandelwal A, Quatrano RS (2009) Isolation and regeneration of protoplasts of the moss *Physcomitrella patens*. Cold Spring Harb Protoc 2009:pdb.prot5140. 10.1101/pdb.prot5140 10.1101/pdb.prot5140 20147070

[CR7] Frangedakis E et al (2021) An Agrobacterium-mediated stable transformation technique for the hornwort model *Anthoceros agrestis*. New Phytol 232:1488–1505. 10.1111/nph.17524 34076270 10.1111/nph.17524 PMC8717380

[CR8] Gao B, Zhang D, Li X, Yang H, Wood AJ (2014) De novo assembly and characterization of the transcriptome in the desiccation-tolerant moss *Syntrichia caninervis*. BMC Res Notes 7:490. 10.1186/1756-0500-7-490 25086984 10.1186/1756-0500-7-490 PMC4124477

[CR9] Gao B, Zhang D, Li X, Yang H, Zhang Y, Wood AJ (2015) De novo transcriptome characterization and gene expression profiling of the desiccation tolerant moss *Bryum**argenteum* following rehydration. BMC Genom. 10.1186/s12864-015-1633-y 10.1186/s12864-015-1633-y PMC4445806 26016800

[CR10] Gao B et al (2017) Desiccation tolerance in bryophytes: the dehydration and rehydration transcriptomes in the desiccation-tolerant bryophyte *Bryum argenteum*. Sci Rep. 10.1038/s41598-017-07297-3 28790328 10.1038/s41598-017-07297-3 PMC5548717

[CR11] Ishida Y, Saito H, Ohta S, Hiei Y, Komari T, Kumashiro T (1996) High efficiency transformation of maize (*Zea**mays* L) mediated by *Agrobacterium tumefaciens*. Nat Biotechnol 14:745–750. 10.1038/nbt0696-745 9630983 10.1038/nbt0696-745

[CR12] Ishizaki K, Chiyoda S, Yamato KT, Kohchi T (2008) Agrobacterium-mediated transformation of the haploid liverwort *Marchantia polymorpha* L., an emerging model for plant biology. Plant Cell Physiol 49:1084–1091. 10.1093/pcp/pcn085 18535011 10.1093/pcp/pcn085

[CR13] Jefferson RA, Kavanagh TA, Bevan MW (1987) GUS fusions: beta-glucuronidase as a sensitive ans versatile gene fusion marker in higher plants. Embo J 6:3901–3907 3327686 10.1002/j.1460-2075.1987.tb02730.x PMC553867

[CR14] Jha P, Shashi RA, Agnihotri PK, Kulkarni VM, Bhat V (2011) Efficient *Agrobacterium*-mediated transformation of *Pennisetum glaucum* (L.) R. Br. using shoot apices as explant source. Plant Cell Tiss Org 107:501–512. 10.1007/s11240-011-0001-0

[CR15] Kumar R, Mamrutha HM, Kaur A, Venkatesh K, Sharma D, Singh GP (2019) Optimization of *Agrobacterium*-mediated transformation in spring bread wheat using mature and immature embryos. Mol Biol Rep 46:1845–1853. 10.1007/s11033-019-04637-6 30707418 10.1007/s11033-019-04637-6

[CR16] Lang D, Zimmer AD, Rensing SA, Reski R (2008) Exploring plant biodiversity: the *Physcomitrella* genome and beyond. Trends Plant Sci 13:542–549. 10.1016/j.tplants.2008.07.002 18762443 10.1016/j.tplants.2008.07.002

[CR17] Li J, Li X, Zhang P (2014a) Micro-morphology, ultrastructure and chemical composition changes of *Bryum argenteum* from a desert biological soil crust following one-year desiccation. Bryologist 117:232–240. 10.1639/0007-2745-117.3.232

[CR18] Li X, Zhang D, Li H, Wang Y, Zhang Y, Wood AJ (2014b) EsDREB2B, a novel truncated DREB2-type transcription factor in the desert legume *Eremosparton songoricum*, enhances tolerance to multiple abiotic stresses in yeast and transgenic tobacco. BMC Plant Biol 14:44. 10.1186/1471-2229-14-44 24506952 10.1186/1471-2229-14-44 PMC3940028

[CR19] Li X, Zhang D, Li H, Gao B, Yang H, Zhang Y, Wood AJ (2015) Characterization of reference genes for RT-qPCR in the desert moss *Syntrichia caninervis* in response to abiotic stress and desiccation/rehydration. Front Plant Sci. 10.3389/fpls.2015.00038 25699066 10.3389/fpls.2015.00038 PMC4318276

[CR20] Liu X, Zhou P, Li X, Zhang D (2021) Propagation of desert moss *Syntrichia caninervis* in peat pellet: a method for rapidly obtaining large numbers of cloned gametophytes. Plant Methods. 10.1186/s13007-021-00740-7 33882971 10.1186/s13007-021-00740-7 PMC8059278

[CR21] Lopez-Obando M et al (2021) The *Physcomitrium* (*Physcomitrella*) *patens* PpKAI2L receptors for strigolactones and related compounds function via MAX2-dependent and-independent pathways. Plant Cell 33:3487–3512. 10.1093/plcell/koab217 34459915 10.1093/plcell/koab217 PMC8662777

[CR22] Manders G, Otoni WC, Vaz FBD, Blackhall NW, Power JB, Davey MR (1994) Transformation of passionfruit (passiflora edulis fv flavicarpa denger) using *Agrobacterium*-*tumefaciens*. Plant Cell Rep 13:697–702. 10.1007/bf00231627 24193523 10.1007/BF00231627

[CR23] Newell CA (2000) Plant transformation technology—developments and applications. Mol Biotechnol 16:53–65. 10.1385/mb:16:1:53 11098468 10.1385/MB:16:1:53

[CR24] Nishimura A, Aichi I, Matsuoka M (2006) A protocol for *Agrobacterium*-mediated transformation in rice. Nat Protoc 1:2796–2802. 10.1038/nprot.2006.469 17406537 10.1038/nprot.2006.469

[CR25] Okada R, Kondo S, Satbhai SB, Yamaguchi N, Tsukuda M, Aoki S (2009) Functional characterization of CCA1/LHY homolog genes, PpCCA1a and PpCCA1b, in the moss *Physcomitrella patens*. Plant J 60:551–563. 10.1111/j.1365-313X.2009.03979.x 19624471 10.1111/j.1365-313X.2009.03979.x

[CR26] Olhoft PM, Flagel LE, Somers DA (2004) T-DNA locus structure in a large population of soybean plants transformed using the *Agrobacterium*-mediated cotyledonary-node method. Plant Biotechnol J 2:289–300. 10.1111/j.1467-7652.2004.00070.x 17134390 10.1111/j.1467-7652.2004.00070.x

[CR27] Proctor MCF, Oliver MJ, Wood AJ, Alpert P, Stark LR, Cleavitt NL, Mishler BD (2007) Desiccation-tolerance in bryophytes: a review. Bryologist 110:595–621. 10.1639/0007-2745(2007)110[595:Dibar]2.0.Co;2

[CR44] Pu X et al (2019) A CRISPR/LbCas12a-based method for highly efficient multiplex gene editing in *Physcomitrella patens*. Plant J 100:863–872. 10.1111/tpj.14478 31350780 10.1111/tpj.14478

[CR28] Raineri DM, Bottino P, Gordon MP, Nester EW (1990) Agrobacterium-mediated transformation of rice (*Oryza-sativa*). Bio-Technol 8:33–38. 10.1038/nbt0190-33

[CR29] Rensing SA, Goffinet B, Meyberg R, Wu S-Z, Bezanilla M (2020) The moss physcomitrium (physcomitrella) patens: a model organism for non-seed plants. Plant Cell 32:1361–1376. 10.1105/tpc.19.00828 32152187 10.1105/tpc.19.00828 PMC7203925

[CR30] Schaefer DG (2002) A newmoss genetics: targeted mutagenesis in *Physcomitrella**patens*. Annu Rev Plant Biol 53:477–501. 10.1146/annurev.arplant.53.100301.135202 12221986 10.1146/annurev.arplant.53.100301.135202

[CR31] Schaefer DG, Zrÿd JP (1997) Efficient gene targeting in the moss *Physcomitrella patens*. Plant J 11:1195–1206. 10.1046/j.1365-313x.1997.11061195.x 9225463 10.1046/j.1365-313x.1997.11061195.x

[CR32] Schuette S, Wood AJ, Geisler M, Geisler-Lee J, Ligrone R, Renzaglia KS (2009) Novel localization of callose in the spores of *Physcomitrella patens* and phylogenomics of the callose synthase gene family. Ann Bot-Lond 103:749–756. 10.1093/aob/mcn268 10.1093/aob/mcn268 PMC2707875 19155219

[CR33] Schuette S, Piatkowski B, Corley A, Lang D, Geisler M (2015) Predicted protein-protein interactions in the moss *Physcomitrella**patens*: a new bioinformatic resource. BMC Bioinf. 10.1186/s12859-015-0524-1 10.1186/s12859-015-0524-1 PMC4384322 25885037

[CR34] Silva AT et al (2021) To dry perchance to live: Insights from the genome of the desiccation-tolerant biocrust moss *Syntrichia caninervis*. Plant J 105:1339–1356. 10.1111/tpj.15116 33277766 10.1111/tpj.15116

[CR35] Stark LR, Brinda JC (2015) Developing sporophytes transition from an inducible to a constitutive ecological strategy of desiccation tolerance in the moss *Aloina ambigua*: effects of desiccation on fitness. Ann Bot-Lond 115:593–603. 10.1093/aob/mcu252 10.1093/aob/mcu252 PMC4343288 25578378

[CR36] Stark LR, McLetchie DN, Eppley SM (2010) Sex ratios and the shy male hypothesis in the moss *Bryum argenteum* (Bryaceae). Bryologist 113:788–797. 10.1639/0007-2745-113.4.788

[CR37] Takata N, Eriksson ME (2012) A simple and efficient transient transformation for hybrid aspen (*Populus**tremula* x *P.**tremuloides*). Plant Methods. 10.1186/1746-4811-8-30 22871142 10.1186/1746-4811-8-30 PMC3476444

[CR38] Tsuboyama S, Kodama Y (2014) AgarTrap: a simplified agrobacterium-mediated transformation method for sporelings of the liverwort *Marchantia polymorpha* L. Plant Cell Physiol 55:229–236. 10.1093/pcp/pct168 24259681 10.1093/pcp/pct168

[CR39] Wu H-Y et al (2014) AGROBEST: an efficient Agrobacterium-mediated transient expression method for versatile gene function analyses in *Arabidopsis* seedlings. Plant Methods. 10.1186/1746-4811-10-19 24987449 10.1186/1746-4811-10-19 PMC4076510

[CR40] Yang X-f, Yu X-q, Zhou Z, Ma W-J, Tang G-x (2016) A high-efficiency *Agrobacterium**tumefaciens* mediated transformation system using cotyledonary node as explants in soybean (*Glycine**max* L.). Acta Physiol Plant. 10.1007/s11738-016-2081-2

[CR41] Zhang X, Henriques R, Lin S-S, Niu Q-W, Chua N-H (2006) Agrobacterium-mediated transformation of *Arabidopsis thaliana* using the floral dip method. Nat Protoc 1:641–646. 10.1038/nprot.2006.97 17406292 10.1038/nprot.2006.97

[CR42] Zhang YM, Chen J, Wang L, Wang XQ, Gu ZH (2007) The spatial distribution patterns of biological soil crusts in the Gurbantunggut Desert, Northern Xinjiang. China J Arid Environ 68:599–610. 10.1016/j.jaridenv.2006.06.012

