# Supplementary material for: A simple, highly efficient Agrobacterium tumefaciens‐mediated moss transformation system with broad applications
Source: aBIOTECH. 2024 Jul 19;5(4):476–87. doi: 10.1007/s42994-024-00174-4 (PMC11624164; doi:10.1007/s42994-024-00174-4)
Supplement: Supplementary file 1 — Supplementary file1 (PDF 377 KB) [file 42994_2024_174_MOESM1_ESM.pdf]

Developing a simple, and high-efficiency Agrobacterium-mediated moss transformation system with broad application

Ping Zhou[1,2], Xiujin Liu[1,3], Yuqing Liang[1,3], Yan Zhang[4,5], Xiaoshuang Li[1,3], Daoyuan Zhang[1,3*]

[1] State Key Laboratory of Desert and Oasis Ecology, Key Laboratory of Ecological Safety and Sustainable Development in Arid Lands, Xinjiang Institute of Ecology and Geography, Chinese Academy of Sciences, Urumqi, 830011, China

[2] Department of Food Science and Engineering, Moutai Institute, Renhuai, 564502, China

[3] Xinjiang Key Lab of Conservation and Utilization of Plant Gene Resources, Xinjiang Institute of Ecology and Geography, Chinese Academy of Sciences, Urumqi 830011, China

[4] University of Chinese Academy of Sciences, Beijing, 100049, China

[5] Shanghai Center for Plant Stress Biology, CAS Center for Excellence in Molecular Plant Sciences, Chinese Academy of Sciences, Shanghai, 200032, China

[*] Correspondence: zhangdy@ms.xjb.ac.cn; Tel.: +86-13899980890

Supplementary Information

Figure of contents

Fig. S1：Selecting protonema one month later in moss.

Fig. S2：PCR validation of transgenic lines

Fig. S3：PCR verification of the heritability of transgenic moss

Fig. S4：Outline of transforming moss protonema or gametophyte.

Fig.S1

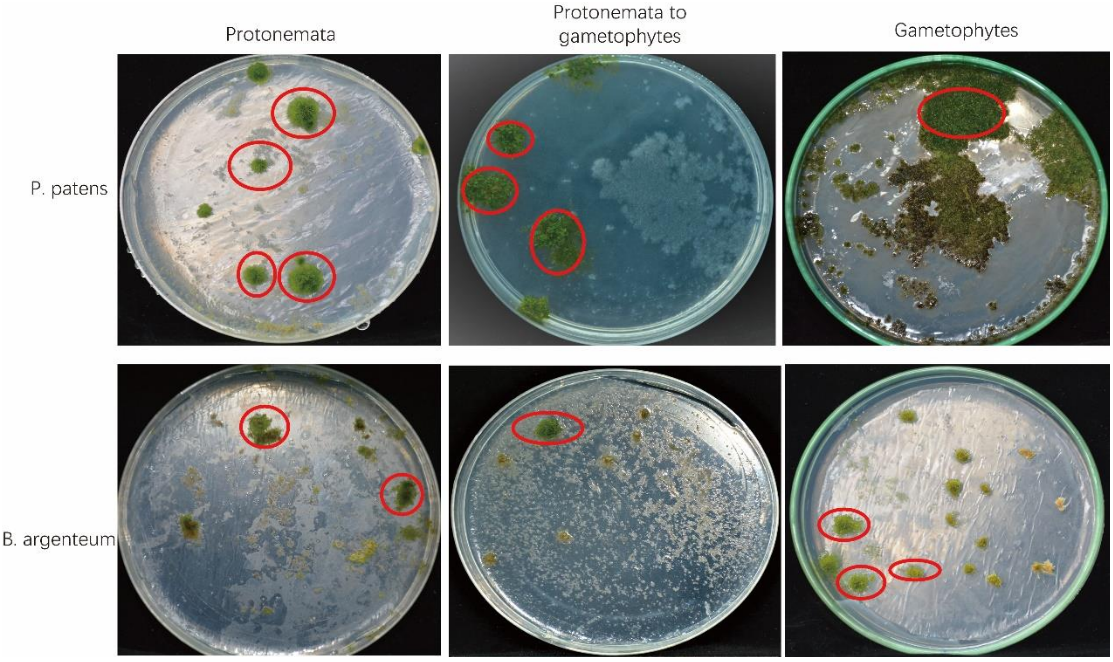

Fig. S1 Selecting protonema one month later in moss. The red cycles represents the surviving moss after screening.

Fig. S2

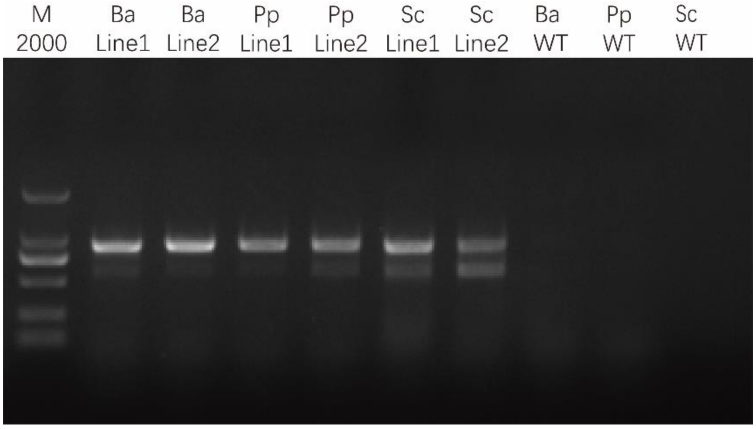

Fig. S2 PCR amplification of GUS fragment gene in hygromycin resistant plants after selection. Ba: *B. argenteum*; Sc: *S. caninervis*; Pp: *P. patens*.

Fig.S3

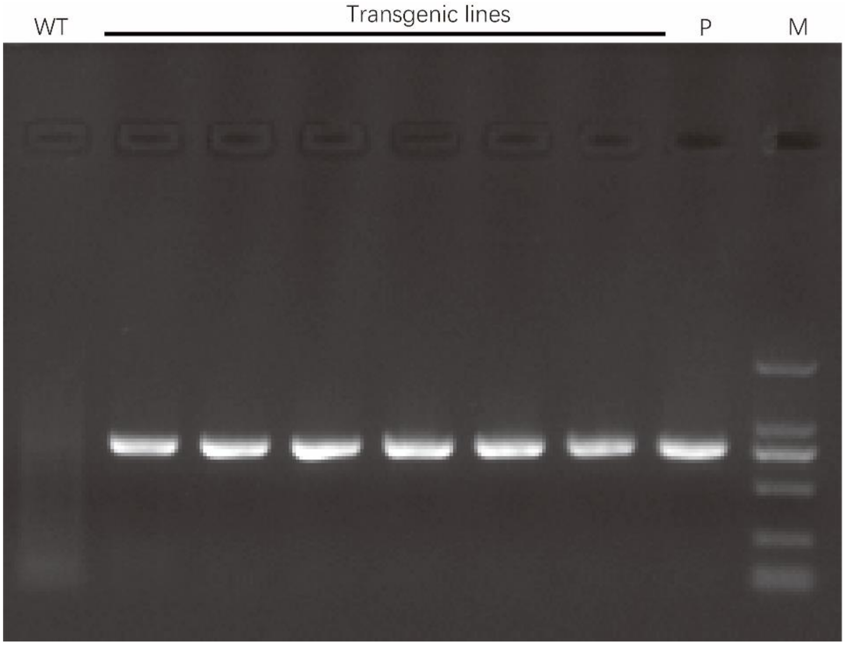

Fig. S3 PCR amplification of *gus*A gene fragment in transgenic moss after 10 generations. WT: wild type; P: plasmid; M: maker 2000.

Fig. S4

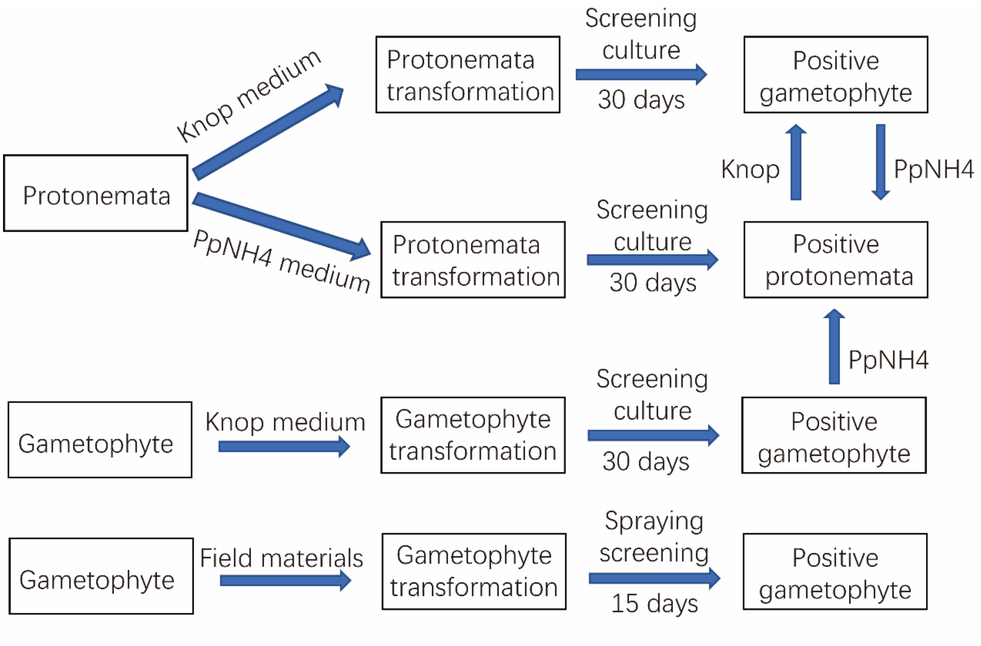

Fig S4 Outline of transforming moss protonema or gametophyte.
